# Study on the status of thyroid function and thyroid nodules in chinese breast cancer patients

**DOI:** 10.18632/oncotarget.20542

**Published:** 2017-08-24

**Authors:** Yanling Shi, Xin Li, Liang Ran, Bilal Arshad, Hao Li, Zhou Xu, Chunxia Zhao, Yutuan Wu, He Wu, Haoran Chen, Hong-Yuan Li, Kai-Nan Wu, Ling-Quan Kong

**Affiliations:** ^1^ Department of Endocrine and Breast Surgery of The First Affiliated Hospital of Chongqing Medical University, Chongqing 400016, China; ^2^ The Medical Examination Center of The First Affiliated Hospital of Chongqing Medical University, Chongqing 400016, China

**Keywords:** breast cancer, thyroid disease, thyroid function, thyroid hormone, thyroid nodule

## Abstract

We performed a study to investigate the status of thyroid nodules and thyroid functions in Chinese breast cancer women. The clinical data of female patients with breast cancer or benign breast diseases and normal populace were evaluated. The thyroxine(T4) level in initially diagnosed breast cancer patients were significantly higher than those in benign breast diseases patients (7.68±1.51 vs 7.29±1.52ug/dl, p<0.001), while the TSH levels were slightly lower than in benign breast diseases patients(3.23±4.59 vs 3.60±6.74uIU/ml, p=0.302). The overall incidence of hypothyroidism in initially diagnosed breast cancer and benign breast diseases patients were 28.65% and 32.74%(p=0.195). During chemotherapy, the T4(7.08±1.69ug/dl), fT3(2.87±0.48pg/ml) and fT4(0.83±0.15ng/dl) levels were significantly lower than in initially diagnosed breast cancer patients(7.68±1.51ug/dl, 3.07±0.50pg/ml, 0.88±0.20ng/dl, p<0.05). The incidence of thyroid nodules in initially diagnosed breast cancer patients, benign breast diseases patients and healthy population were 56.17%, 43.64%, 34.49%(p<0.001). The incidence of TI-RADS≥4 TN in initially diagnosed breast cancer patients and benign breast diseases patients were significantly higher than in normal population(7.27% vs 9.45% vs 2.87%, p<0.001). The incidence of TI-RADS≥4 thyroid nodules in breast cancer patients receiving chemotherapy was significantly higher than in initially diagnosed breast cancer patients(11.71% vs 7.27%, p<0.05). These data indicate that the incidence of thyroid disease in breast disease patients is higher than in normal population in China, and the breast diseases, especially breast cancer, might be related to the high incidence of thyroid nodules.

## INTRODUCTION

Breast cancer (BC) is the most common cancer among women globally and ranks as the second leading cause of cancer death among women (after lung cancer) and, by far is the second common cancer in the world [1]. In recent years, the BC incidence is rising rapidly in developing countries as a result of changes in reproductive risk factors, dietary habits and increasing life expectancy [2]. And the BC mortality rate also increased significantly and the average age of BC patients was quite early compared to western populace.

Thyroid disease is a common clinical hyperplastic disease with 2%-5% being malignant. It is predicted that papillary thyroid cancer will become the third most common cancer in women in the United States by 2019. Meanwhile, and the prevalence of thyroid nodules(TN) has increased significantly in China in recent years [3]. Both breast and thyroid gland are hormone sensitive organs. It has long been recognized that estrogen is a risk factor for BC. Recent studies have found that estrogen is also a potent growth factor both for benign and malignant thyroid cells that may explain the difference in the prevalence of thyroid nodules and thyroid cancer [4]. Thyroid hormones (TH) play a major role in physiological processes crucial for growth, maturity, and metabolism. It has therefore been suggested that TH (including triiodothyronine, T3/thyroxine, T4/free thyroxine, fT4) and low pre-diagnostic thyroid peroxydase antibody(TPO-Ab) levels are associated with BC risk, and TH could further stimulate tumor growth [5–8]. Nina Ditsch et al. revealed specific alterations in the expression of Thyroid receptors(TRs) in breast cancer patients. Significant correlations of the expression of TRs were found with further prognostic histopathological parameters such as tumor size, axillary lymph node involvement, grading and hormone receptor status [9].However, the relationship between thyroid disease and breast disease was unknown. In this study, we aimed to perform a study to investigate the status of thyroid function and thyroid nodules in Chinese breast cancer patients.

## RESULTS

The T4 level in initially diagnosed BC patients were significantly higher than those in BBD patients(7.68±1.51 vs 7.29±1.52ug/dl, p<0.001), while the TSH levels were slightly lower than in BBD patients (3.23±4.59 vs 3.60±6.74uIU/ml, p=0.302). The T3, fT4 levels of initially diagnosed BC patients was higher than in BBD patients, but without significant difference. The overall incidence of hypothyroidism in BBD and initially diagnosed BC patients were 32.74% and 28.65%, respectively (p=0.195). (Table 1 and Table 2).

**Table 1:** Comparison of thyroid function status among initially diagnosed breast cancer patients, benign breast disease patients and breast cancer during chemotherapy ($\bar{x}\pm s$)

Groups	case	Age	T3(ng/ml)	T4(ug/dl)	FT3(pg/ml)	FT4(ng/dl)	TSH(uIU/ml)
Initially diagnosed breast cancer	726	51.03±10.98	1.05±0.22	7.68±1.51	3.07±0.50	0.88±0.20	3.23±4.59
benign breast diseases	336	44.5±12.23^*^	1.04±0.22	7.29±1.52^**^	3.07±0.43	0.86±0.14	3.60±6.74
during chemotherapy	153	49.03±8.50^*^	1.04±0.24	7.08±1.69^**^	2.87±0.48^**^	0.83±0.15^*^	2.77±3.18

^*^p<0.05 ^**^ p<0.001 vs. initially diagnosed breast cancer.

**Table 2:** Comparison of the incidence of hypothyroidism among initially diagnosed breast cancer patients, breast benign diseases patients and patients during chemotherapy

Groups	case	Age	Incidence of clinical hypothyroidism	Incidence of subclinical hypothyroidism	Incidence of Low T3 syndrome	Incidence of total hypothyroidism
Initially diagnosed BC patients	726	51.03±10.98	2.2%(16/726)	20.25% (147/726)	6.2% (45/726)	28.65% (208/726)
BBD patients	336	44.5±12.23^*^	2.38% (8/336)	24.4% (82/336)	5.95% (20/336)	32.74% (110/336)
during chemotherapy	153	49.03±8.50^*^	2.61% (4/153)	21.57% (33/153)	6.54% (10/153)	30.72% (47/153)

^*^p<0.05 vs. initially diagnosed breast cancer.

During chemotherapy, the T4(7.08±1.69ug/dl), fT3(2.87±0.48pg/ml) and fT4(0.83±0.15ng/dl) levels of BC patients receiving chemotherapy were significantly lower than those in initially diagnosed BC patients(7.68±1.51ug/dl, 3.07±0.50pg/ml, 0.88±0.20ng/dl, p<0.05). Similar trend was observed in TSH, T3 levels but the difference was not found to be statistically significant. No statistical difference was observed in the total incidence of hypothyroidism during chemotherapy (30.72%) and among initially diagnosed BC patients (28.65%, p=0.625).(Table 1 and Table 2).

The incidences of TN in normal populace, BBD patients and initially diagnosed BC patients were 34.49%, 43.64% and 56.17% (p<0.001). Among the age-standardized incidence rate of above groups, the incidences of TN in initially diagnosed BC group and BBD group were also higher than in normal population. In the age groups(∼39, 40∼49), the incidences of TN in initially diagnosed BC group were significant higher than in BBD group. But in the age group (50∼59, 60∼), the incidence of TN in initially diagnosed BC group was lower than in BBD, with no significant statistical difference. The incidences of TN increased with aging in the groups of normal population, BBD and initially diagnosed BC patients. The incidences of TN in BC patients during chemotherapy and after systemic therapy were 52.93% and 55.79%, respectively, with no significant statistical difference (p<0.519).(Table 3 and Table 4).

**Table 3:** Comparison of the status of thyroid nodules among the normal population, initially diagnosed breast cancer patients and breast benign disease patients

	case	Age	incidence of thyroid nodules	incidence of thyroid nodules with TI-RADS≥4
Normal population				
Total	14161	15≦Age≦97	34.49%^**^(4884/14161)	2.87%^**^(406/14161)
	6977	Age<40	20.64%^*^(1440/6977)	1.50%^*^(107/6977)
	3585	40≦Age<50	39.36%^**^(1411/3585)	3.29%^**^(118/3585)
	2251	50≦Age<60	51.13%^*^(1151/2251)	4.22%(95/2251)
	1348	Age≥60	65.43%(882/1348)	6.38%(86/1348)
Initially diagnosed BC patients				
Total	632	21≦Age≦88	56.17^Δ^ (355/632)	7.27%(46/632)
	84	Age<40	35.71%^Δ^ (30/84)	5.95%(5/84)
	242	40≦Age<50	54.13%^Δ^ (131/242)	7.85%(19/242)
	175	50≦Age<60	59.43%(104/175)	6.86%^Δ^ (12/175)
	131	Age≥60	68.70%(90/131)	7.63%(10/131)
BBD patients				
Total	275	17≦Age≦86	43.64%^▲^ (120/275)	9.45%^▲▲^ (26/275)
	61	Age<40	16.39%(10/61)	3.28%(2/61)
	132	40≦Age<50	40.15%(53/132)	7.58%^▲^ (10/132)
	54	50≦Age<60	66.67%^▲^ (36/54)	18.52%^▲^ (10/54)
	28	Age≥60	75.00%(21/28)	14.29%(4/28)

*p<0.05 **p<0.001 vs. initially diagnosed BC group^Δ^ p<0.05, ^Δ^p<0.001 vs Benign breast disease group ^▲^p<0.05 ^▲▲^p<0.001 vs. normal population.

**Table 4:** Comparison of the status of thyroid nodules among initially diagnosed breast cancer patients, breast cancer patients during chemotherapy and breast cancer after systemic therapy

Groups	case	Age	incidence of thyroid nodules	incidence of thyroid nodules of TI-RADS≥4
initially diagnosed BC patients	632	50.16±10.76	56.17% (355/632)	7.27%^*^(46/632)
BC patients during chemotherapy	461	49.34±8.85	52.93% (244/461)	11.71%^Δ^ (54/461)
BC patients after systemic therapy	233	51.53±9.73	55.79% (130/233)	6.87% (16/233)

^*^p<0.05 vs. breast cancer during chemotherapy ^Δ^p<0.05 vs. breast cancer after systemic therapy.

The incidence of TI-RADS≥4 TN in healthy population was significantly lower compared to BBD patients(2.87% vs 9.45%, p<0.001) and initially diagnosed BC patients (2.87% vs 7.27%, p<0.001). Among the age-standardized incidence rate of the above groups, the incidence of TI-RADS≥4 TN in initially diagnosed BC group and BBD group were higher than in normal population, but no significant difference was observed between initially diagnosed BC patients and BBD patients. However, the small sample size precluded any definitive conclusions among every age groups. The incidence of TI-RADS≥4 TN in BC patients during chemotherapy was higher than in initially diagnosed BC patients(11.71% vs 7.27%, p=0.014); the incidence in BC patients post systemic therapy was lower than in patients under chemotherapy(6.87% vs 11.71%, p=0.046), but the difference between BBD patients and initially diagnosed BC patients was not statistically significant (p=0.883). (Table 3 and Table 4).

## DISCUSSION

Nowadays, a large number of studies have investigated the association between TH levels and the occurrence of BC. A study has found that aging results in a decrease in TSH and T3 levels. Whereas, serum fT4 levels usually remain unchanged [10]. In our study, the mean age of initially diagnosed BC patients were significantly older compared to BBD patients. But the T4, T3, fT4 levels in patients with initially diagnosed BC patients were higher than those in BBD patients, suggesting that high TH levels might be associated with increased risk of BC. Instead, the TSH levels were lower in initially diagnosed BC patients, which could be explained by the negative feedback regulation of higher thyroid function on TSH secretion. According to another research by Rasool, M et al, a significant raise in T3 and T4 levels were observed among BC patients compared to normal controls [11], which is in accordance to our study results. Some studies have revealed that higher fT4 and T3 were significantly associated with an increased risk of BC. While TSH was not associated with increased BC risk [12–13]. But a meta-analysis illustrated that thyroid dysfunction may not be related to increased risk of breast cancer [14]. In our study, the total incidence of hypothyroidism in initially diagnosed BC patients were lower than in BBD patients. Another study suggested that primary hypothyroidism was associated with a reduced risk of primary BC and a more invasive disease [15]. Suggesting that hyperthyroidism have a significant role in BC cell proliferation. But on the contrary, HUANG ed al revealed that the incidence of hypothyroidism in initially diagnosed BC were higher than in BBD patients, and fT3 levels were lower than in BBD patients, which suggests that hypothyroidism might cause an increased BC risk [16]. All of the above results indicate that breast cancer and thyroid functions are probably related to each other, but it still need further investigation.

Chemotherapy is one of the most effective means in treating cancer. It's a kind of systemic therapy which not only kills cancer cells, but also damage the normal cells. So, chemotherapy may cause thyroid injury and change thyroid function. In our study, the mean age of initially diagnosed BC patients were significantly older compared to BC patients receiving chemotherapy, but the T4, fT3 and fT4 levels were significantly lower in BC patients receiving chemotherapy, and similar trend was also observed in TSH, T3 levels without significant difference. The TH levels declined in patients receiving chemotherapy can be explained by the fact that the thyroid cells were damaged by chemicals, thus reducing TH secretion. The TSH level decreased after chemotherapy suggested that the chemicals might inhibit hypothalamic-pituitary-thyroid axis, thus increases the susceptibility of thyroid dysfunction during chemotherapy. Paclitaxel is a chemotherapy drug commonly used for BC treatment, which should be used with dexamethasone. A small sample size study suggested that short-term administration of pharmacological doses of glucocorticoids suppress the secretion of TSH via direct effect on the anterior pituitary gland [17].

TN is a common disease, especially in regions with inadequate iodine supply, with 2%-5% odds of malignancy. Yao Liu et al. conducted a large scale cross-sectional survey with 67,781 residents (33,020 men, 34,761 women), in Shanxi, China, showed that approximately 30.7% of men and 39.9% of women in Northwest China had TN [3]. In our study, the incidence of TN in normal population (34.49%) was close to that of women in Shanxi, which was significantly lower than those in BBD patients and initially diagnosed BC patients(43.64%, 56.17%, p<0.001), suggested that the breast diseases, especially BC, might be related to the high incidence of TN; and the metabolic activity of BC cells might further stimulate the formation of TN. It is significantly evident from a meta-analysis that patients with autoimmune thyroiditis have increased risk of acquiring BC in addition to increased risk of acquiring anti-thyroid antibodies [18].Meanwhile, another mata-analysis revealed that goiter is also associated with breast disease [19]. Turken et al carried out a study reported that the incidence of goiter was significantly higher in BC patients than in control individuals [20]. These results indicate that breast cancer and thyroid disease are probably related. In this study, the incidence of TN with TI-RADS≥4 in initially diagnosed BC patients was significantly lower than BBD patients, and the TSH levels in initially diagnosed BC patients(3.23±4.59uIU/ml) was lower than BBD patients(3.60±6.74 uIU/ml). TSH stimulates TN growth, which might be one of the reasons for higher incidence of TN in BBD patients than initially diagnosed BC patients, but it also needs to be further investigated.

Currently there have been no studies on the effect of chemotherapy in TN. In this study, the incidences of TN of patients receiving chemotherapy decreased than those initially diagnosed BC patients, which might be due to the result of the chemicals inhibiting hypothalamic-pituitary-thyroid axis leading to the decrease in TSH secretion, thus resulted in decline of TN incidence. Meanwhile, our study suggested that the TSH level of during chemotherapy(2.77±3.18uIU/ml) was lower than in initially diagnosed BC patients(3.23±4.59uIU/m), which supported the above viewpoint. The incidence of TN with TI-RADS≥4 in BC patients receiving chemotherapy increased might be the reason that chemotherapy drugs may cause distortion of TN. But the decline in the incidences of TN of TI-RADS≥4 post chemotherapy might be due to decreased drug stimulation.

In summary, we observed that the incidence of TN and Thyroid dysfunction were higher in breast disease patients, especially in BC patients. E Izkhakov et al, identified that TC patients as a high-risk group [21], and the increased incidence of thyroid cancer will create a heavy burden on the health care system. hence more studies need to be done on this arena and more emphasis need to be laid on screening TN and thyroid function in breast disease patients especially BC patients.

## MATERIALS AND METHODS

### Study population

The study was performed at the Breast Cancer Center of Chongqing and the Medical Examination Center of the First Affiliated Hospital of Chongqing Medical University. It was approved by The Ethics Committee of the First Affiliated Hospital of Chongqing Medical University and had been registered in ChiCTR (Registration number: ChiCTR-OPC-15007289). All the female patients with primary breast disease who received treatment at the Breast Cancer Center of Chongqing from October 2015 to February 2017. This cancer center is one of the largest in southwest China(with approximately 31.4 million people who live in about 82,402.95 km^2^ area).

We performed a study consisting of female breast disease patients who underwent thyroid ultrasonography, including 275 cases of BBD, 632 cases of initially diagnosed BC patients, 461 cases of BC patients receiving chemotherapy and 233 cases of BC patients post-systemic therapy followed-up in our Clinic. At the same time, the data of 14161 normal subjects were comparatively analyzed.

The study also consisting of female breast disease patients who underwent thyroid function and antibody, including 726 cases of initially diagnosed BC patients, 153 cases of BC patients receiving chemotherapy and 336 cases of BBD females.

### Data collection

To investigate thyroid nodules, the size(length, width and depth), location, number, echogenicity, boundary and cystic component were collected and recorded. The ultrasound images of TN were categorized based on Thyroid Imaging and Reporting System (TI-RADS) criteria. Thyroid function was evaluated by measuring the TH including thyroid stimulating hormone (TSH), triiodothyronine(T3), free triiodothyronine(fT3), thyroxine (T4), free thyroxine(fT4), thyroglobulin antibody(TGAb), thyroid peroxidase antibody(TPOAb), thyrotrophin receptor antibody(TRAb) levels. All the above TH parameters were measured by electro-chemiluminescence immunoassay method and performed in the laboratory of the First Affiliated Hospital of Chongqing Medical University. The benign and malignant diseases were diagnosed by the Pathology Center of Chongqing Medical University. Also the chemotherapy for BC was carried out in accordance with the standard chemotherapy regimens.

### Statistical analysis

The SPSS Statistics 22 and Word Processing System EXCEL 2016 were used for statistical analysis. The categorical parameters of the incidence of TN and TN of TI-RADS≥4 were evaluated with Chi-square test/Chi-Square Goodness-of-Fit Test and P<0.05(95% CI) was considered to be statistically significnat. The TH levels were expressed by Mean±standard deviation (x¯±s) and analyzed with independent-samples T test or non-parametric rank-sum test for unequal variances. α=0.05 was considered as test reference level. The mean age was derived as mean±standard deviation (x¯±s), P<0.05 was considered to be statistically significant.

## Abbreviations

BC: Breast Cancer
TD: Thyroid Disease
TH: Thyroid Hormone
TN: Thyroid Nodule
BBD: Breast Benign Disease
TSH: thyroid stimulating hormone
T3: triiodothyronine
fT3: free triiodothyronine
T4: thyroxine
fT4: free thyroxine
TGAb: thyroglobulin antibody
TPOAb: thyroid peroxidase antibody
TRAb: athyrotrophin receptor antibody.
